# Polarized Macrophages in Periodontitis: Characteristics, Function, and Molecular Signaling

**DOI:** 10.3389/fimmu.2021.763334

**Published:** 2021-12-07

**Authors:** Xiaoyu Sun, Jike Gao, Xiang Meng, Xiaoxuan Lu, Lei Zhang, Ran Chen

**Affiliations:** Key Laboratory of Oral Diseases Research of Anhui Province, Department of Periodontology, Stomatologic Hospital & College, Anhui Medical University, Hefei, China

**Keywords:** periodontitis, macrophages, polarization, cytokinea, bone resorption

## Abstract

Periodontitis (PD) is a common chronic infectious disease. The local inflammatory response in the host may cause the destruction of supporting periodontal tissue. Macrophages play a variety of roles in PD, including regulatory and phagocytosis. Moreover, under the induction of different factors, macrophages polarize and form different functional phenotypes. Among them, M1-type macrophages with proinflammatory functions and M2-type macrophages with anti-inflammatory functions are the most representative, and both of them can regulate the tendency of the immune system to exert proinflammatory or anti-inflammatory functions. M1 and M2 macrophages are involved in the destructive and reparative stages of PD. Due to the complex microenvironment of PD, the dynamic development of PD, and various local mediators, increasing attention has been given to the study of macrophage polarization in PD. This review summarizes the role of macrophage polarization in the development of PD and its research progress.

## Introduction

PD is a chronic infectious disease caused by the chronic destruction of supporting periodontal tissue initiated by plaque biofilms and is characterized by microbial-related and host-mediated inflammation, leading to the loss of periodontal attachment (1). The latest estimates show that there are approximately 3.5 billion people in the world with untreated caries, severe periodontitis, tooth loss (2), and more than 10% of adults in the world may be affected by severe PD (3, 4). Currently, it is believed that the infection mechanism of PD includes the direct spread of infection, the spread of bacteria into the circulation, and the immune/inflammatory response of the body caused by periodontal pathogenic bacteria and their products. These mechanisms trigger the host defense mechanism, which can increase the inflammatory load of the whole body, making PD closely related to general health as well (5, 6). Periodontal inflammation is caused by the confrontation between pathogenic microorganisms in the subgingival biofilm and various immune cells in the tissue, of which polymorphonuclears can enter the oral mucosa through highly permeable epithelial cells (7). Other immune cells include T cells, B cells, innate lymphocytes, and macrophage dendritic cells (8). The defense response of the host has a dual role in PD, and further immunopathological research will help to identify new clinical treatment strategies.

**Figure 1:**
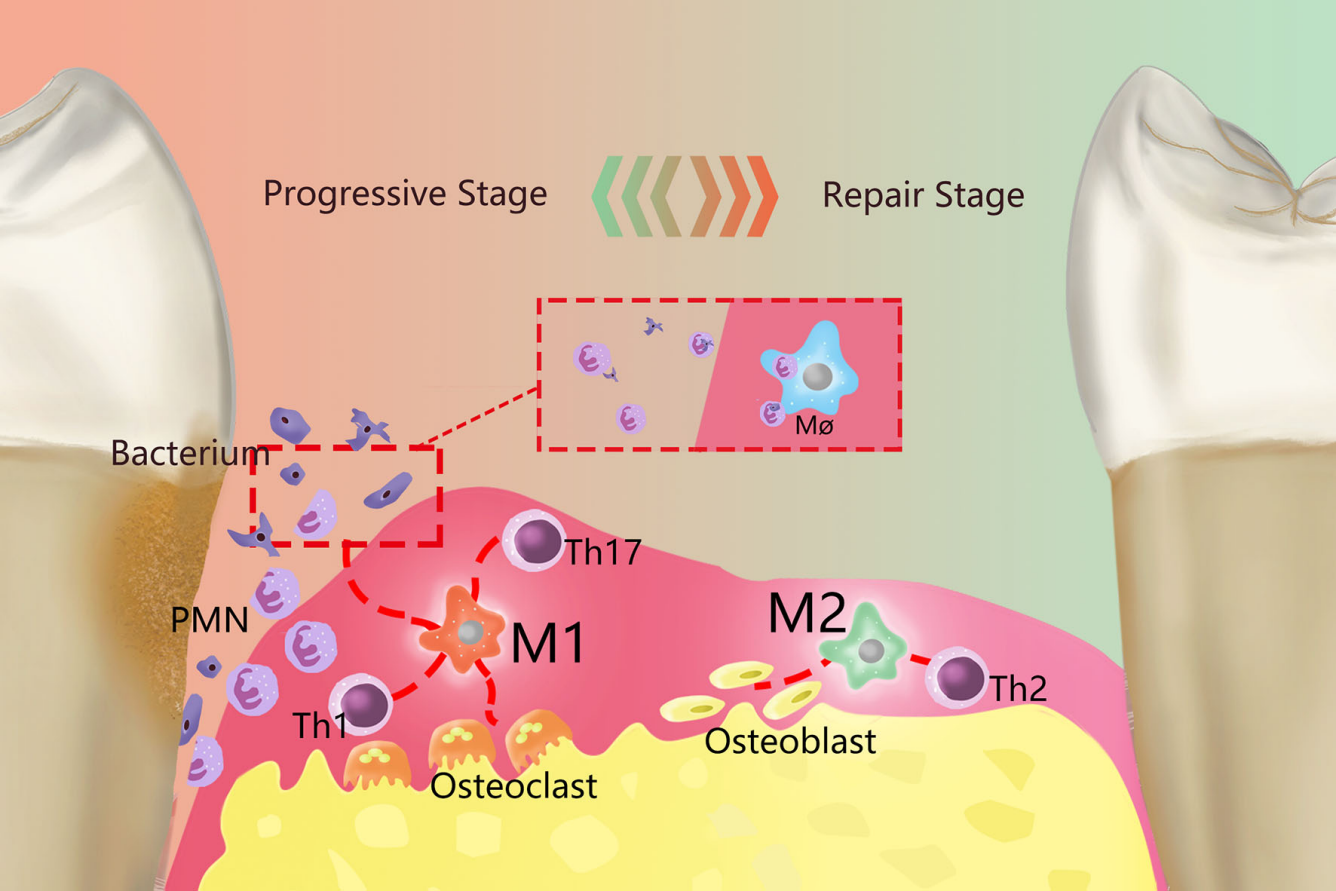
Overview of the role of polarized macrophages in the occurrence and development of PD. Resident macrophages in PD generate two primary phenotypes, M1 and M2, through polarization, which dominate the developmental and regression stages of inflammation, respectively. M1 is primarily proinflammatory and produce a series of proinflammatory factors and work together with Th1 cells, Th2 cells and other cells. Primarily by working with Th1-type immune cells, M1 can remove periodontal pathogenic microorganisms by recruiting PMNs. Meanwhile, M1 activates osteoclasts and causes absorption of the alveolar ridge. M2 primarily plays an anti-inflammatory role and are mainly immune to Th2 cells. M2 terminates inflammatory progression by promoting the apoptosis of M1and PMNs, performed tissue repair through various anti-inflammatory factors, and can activate osteoblasts to restore bone tissue.

In periodontal tissues, macrophages make significant contributions to tissue homeostasis and defense (9), but excessive aggregation and activation of macrophages can also lead to periodontal tissue damage, bacteria and their products (such as endotoxin, etc.) can activate the monocyte/macrophage system and produce a large number of proinflammatory factors that can cause inflammation or an immune response (10). At the same time, due to the increased activity of monocytes and osteoclasts, periodontal supporting tissue will be destroyed and absorbed. Correspondingly, PD is also an inflammatory osteolytic disease (11). The process by which macrophages exhibit different functional phenotypes in response to different stimuli is called polarization (12). Polarized macrophages are primarily divided into two categories according to their functions: M1 macrophages (M1) are primarily involved in the Th1-type immune response, while M2 macrophages (M2) are primarily involved in the Th2-type immune response (13, 14). The polarization of macrophages exists in a dynamic equilibrium during the development of inflammation, and M1 and M2 are the two extreme phenotypes of the dynamic balance between proinflammatory and anti-inflammatory functions in the macrophage polarization spectrum (**Figure 1**). The M1/M2 ratio can provide useful information concerning the health of periodontal tissues (9); for example, during orthodontics, an increased M1/M2 ratio can lead to alveolar bone resorption, while a decreased M1/M2 ratio can inhibit bone resorption (15). Therefore, it is necessary to clarify the function of M1 and M2 in PD pathology.

Currently, understanding of the role of macrophages in PD is limited. In recent years, research on the role of macrophage polarization in the occurrence and development of PD has gradually increased. In this study, a variety of cytokines, enzymes and other effectors related to the polarization of macrophages in periodontal tissues are summarized, and the research results and reviews of periodontal and other macrophage-related inflammatory diseases are cited, highlighting the mechanism and function of the polarization of macrophages in periodontal tissues.

## Overview of Periodontitis

PD is an infectious inflammatory disease related to an imbalance in the microflora and its prevalence increases with age. Its clinical manifestations primarily include the irreversible destruction of tooth supporting tissues and the formation of periodontal pockets, which is the primary cause of tooth loss in adults. PD is one of the most common ailments observed in dental clinics (16). In addition, there is a close relationship between PD and systemic diseases (17), such as type 2 diabetes, cardiovascular disease, rheumatoid arthritis, etc. (18), supporting the relationship between periodontal disease and systemic diseases as both infectious and inflammatory (19). PD comprises a group of destructive periodontal diseases with different clinical manifestations, different responses to treatment, different progress rates and different laboratory findings. Currently, the clinical pathology of PD is not fully understood.

The primary pathological feature of PD is inflammation from the gingival to the periodontal ligament of deep periodontal tissues, alveolar bone, and even cementum involvement (20). According to the histopathological manifestations, PD can be divided into an active stage (progressive stage) and a static stage (repair stage) (21). The chemotaxis and removal ability of neutrophils and monocytes directly affects the body’s ability to defend against periodontal microbial infection (22). When in contact with foreign bacteria, macrophages increase their bactericidal capacity and stimulate the antimicrobial response of other cells by secreting cytokines (23). However, due to the diverse functions of cytokines, macrophages also play an important role in stimulating tissue destruction (24). The study of various macrophage polarization phenotypes is helpful for further explaining the pathological changes that occur in PD.

## Overview of Macrophages in Periodontal Tissue

Macrophages (Møs, Macs) are innate immune cells on the surface of the epithelium that quickly respond to infection. The epithelium of periodontal tissue is highly permeable, and interactions occur between macrophages and the oral environment. Macrophages exhibit distinct responses to different stimuli from the pathogen and symbiotic bacteria and are also the key cells in chronic inflammation-related pathology (25), and their important characteristics include their adaptation to different tissue microenvironments and responses to different pathogenic injuries, consistent with the extremely diverse characteristics of chronic inflammatory responses (10). In addition to resident macrophages in tissues, circulating monocytes differentiate into macrophages through CSF-1 or IL-34 binding receptor CSF-1R in tissues and also participate in osteoclast differentiation (26). The expression of CSF-1 is increased in the gingival tissues of patients with periodontitis (27). Macrophages and neutrophils can be recruited to junctional epithelium and other periodontal tissues by keratinocyte-derived chemokines, macrophage inflammatory protein-2, and other chemotactic and adhesion factors, and this process is not affected by bacterial flora (28).

Macrophages play a role in defending against bacterial infection, antigen presentation, mobilization and regulation of the immune defense response, maintaining the balance between the host and microorganisms. Macrophages are efficient antigen-presenting cells that are especially good at stimulating T cells (29), functioning by phagocytosing pathogens (30), engulfing (31) and secreting cytokines to amplify the immune system, induce and expand inflammation, catalyze the local inflammatory response and stimulate tissue destruction. Macrophages can recognize pathogen-associated molecular patterns (PAMPs) through Toll-like receptors and generate specific cytokines and chemotactic molecules to recruit non-resident neutrophils and other white blood cells to participate in the defense response (32). Macrophages also play a valuable role in the process of terminating inflammation and tissue repair; for example, by sensing phosphatidylserine to recognize apoptotic cells (ACs) (33), macrophages can phagocytose and remove apoptotic or dying PMNs to prevent the uncontrolled release of enzymes by dead or overactivated PMNs to prevent further exacerbation of inflammation (**Figure 1**). The gradual death of neutrophils along with the formation of neutrophil extracellular fibers (NETs) modified with antibacterial proteins is known as NETosis, distinct from apoptosis and necrosis. Nakazawa et al. (22) found *in vitro* that both M1 and M2 could recognize the decomposition of NETs, but M2 played a proinflammatory role in the early stage of the reaction, and proinflammatory cytokines/chemokines were secreted in the supernatant. The M1 group had earlier undergone cell death with nuclear decondensation, with increased levels of M1-derived DNA in the supernatant. Nakazawa et al. believed that m1-derived extracellular DNA after NETs decomposition could assist PMN to generate NETs, thus enhancing the antibacterial effect (34), and the early proinflammatory response of M2 was also one of the host defense mechanisms. *In vitro* studies (23), Haider et al. found that macrophages with a proinflammatory phenotype can improve the ability to clear NETs by secreting DNA enzymes and pinocytosis. Members of the red and orange complex commonly produce active DNA enzymes in late periodontitis, which may be one of the mechanisms to escape the killing effects of NETs.

In addition to phagocytosis and interaction with PMN, macrophages also play distinct roles during different stages of PD through polarization. PD is associated with enhanced M1 and M2 phenotypes in macrophages, and the conversion of the M2 to M1 phenotype may be a key mechanism of mediating periodontal tissue injury (35). The early development of PD-related periodontal tissue infiltration of macrophages is increased, prioritizing M1, meanwhile the proportion of M1 and periodontal inflammation activity progression are positively correlated; in the PD stationary phase, M2 related factor expression, Th2 and Treg cell immunosuppression and repair effects are increased (35, 36). In ligation-induced experimental animal models of PD (37), Viniegra et al. found that high mRNA levels of TGF-β, CD80, and TNF-α were present during the early inflammatory process, indicating active metabolism of M1, while high levels of CD206 mRNA were observed during tissue healing, suggesting massive proliferation of the M2 phenotype. The polarization of macrophages is induced and regulated by various microenvironmental signals (38). Polarized macrophages play regulatory and inductive roles in different stages of PD by expressing different products. The M1 and M2 transition is an important mechanism for the transition between active and inactive PD.

Macrophages were also associated with an increase in the prevalence of periodontitis with age. Studies on mouse bone marrow derived macrophages have found that macrophage polarization also changes with age (39). The possible mechanisms of macrophages and inflammatory aging include accumulation of damage/danger associated molecular patterns (DAMPs), decreased phagocytosis, and decreased ability to induce osteoblasts (40). In novel coronavirus/COVID-19 *in vitro* mice, Duarte et al. stimulated M-CSF and IL-34-induced macrophages with spike proteins, showing age and gender dependent effects (41). Clark et al. found that expressions of M1-related markers and proinflammatory cytokines and chemokines were significantly increased in elderly mice (42); found in their follow-up study of periodontal tissue, while elderly group and young group of mice in different period, there was no significant difference between the number of macrophages, but the elderly mice depleted macrophages more quickly in recovery stage (43). Although the depletion of macrophages stops inflammation earlier, it remains to be seen whether the process of tissue rebuilding and healing will also stop earlier.

The polarization and mechanisms of action of two representative phenotypes of M1/M2 macrophages will be reviewed in the following sections.

## Polarization of Macrophages in Periodontitis and Its Role in Periodontitis Pathophysiology

Macrophages exhibit high heterogeneity and plasticity. Different tissue and organ distributions or local microenvironment changes, or even *in vitro* stimulation differences, lead to different immune responses from macrophages and result in differential functional phenotypes (10). The primary sources of tumor necrosis factor-α (TNF-α), interleukin-1β (IL-1β), interleukin-6 (IL-6), prostaglandin E2 (PGE2) and matrix metalloproteinases-1 (MMPs-1) in cytokines and effector molecules related to periodontal tissue destruction are monocytes/macrophages (**Table 1**). Below, some recent studies regarding macrophage polarization and PD are highlighted.

**Table 1:** The polarization types, characteristics and basic functions of macrophages.

Phenotypes		Stimuli	Special surface receptor	Express product	Main function
M1		LPS, TNF-α, IFN-Y, GM-CSF	MHC II, CD86, CD80	IL-12, IL-23, IL-10, IL-1, TNF, IL-6, product of iNOS	Th1 responses; type I inflammation; tumor resistance
M2	M2a	IL-4 and IL-13	MHC II, CD206, SRs	Polyurethane of Arg, IL-10, decoy IL-1R, CCL17	Th2 responses; type II inflammation
	M2b	IC + TLR/IL-1R agonists	MHC II, CD86	IL-10, IL-12, TNF, IL-1, IL-1, IL-6	Th2 activation; immunoregulation
	M2c	IL-10, Glucocorticoids	MHC II, CD206	IL-10, TGF-β, MerTK	immunoregulation; matrix deposition and tissue remodeling; phagoticing apoptotic cell

M1 are usually induced by LPS or certain cytokines (such as IFN-γ, TNF-α, and GM-CSF) and have antigen-presenting ability. It produces high levels of proinflammatory cytokine production (IL-1β, IL-6, IL-12, and IL-23), CCXL9, and low levels of IL-10. M2 can be polarized by cytokines (IL-4, IL-10, and IL-13), glucocorticoids, immune complexes, etc. Different stimulus factors can induce different subtypes of M2, such as M2a (wounding healing macrophages), M2b (regulatory macrophages), M2c (acquired deactivation macrophages), M2d (tumor-associated macrophage) and etc.; these cells are characterized by high levels of IL-10, TGF-β and vascular endothelial growth factor, and low levels of IL-12, tumor necrosis factor-A and IL-1B production (38, 44).

### The Functional Role of M1 in Periodontitis

**Figure 2:**
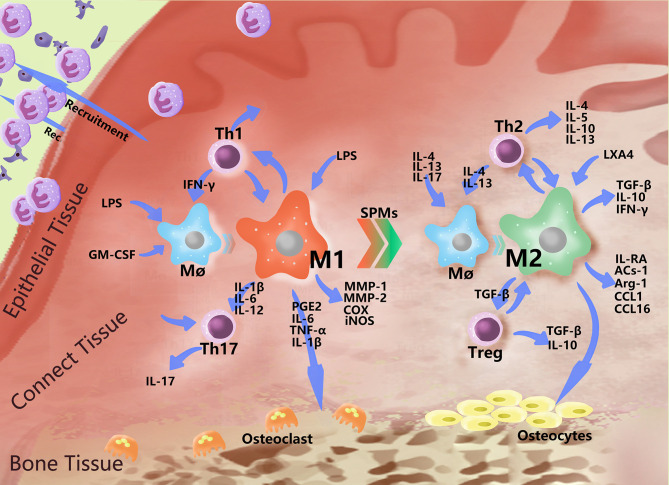
Polarization-related effector molecules in periodontal macrophages. In addition to basic phagocytosis and signal delivery, macrophages polarize in response to various stimuli and express a variety of effector molecules. The figure indicates some effector molecules involved in this role. In PD, macrophages recruit neutral polynucleated granulocytes to fight bacteria near the gingiva, mutually promoting the Th1 immune response and causing damage to bone tissue by stimulating Th17 cells and producing PGE2 and MMPs. When the level of bacteria decreases, the number of proinflammatory mediators increase, and the immunomodulatory effects of M2 began to play a dominant role, interacting with Th2 to produce a series of anti-inflammatory molecules, which begin to repair the periodontal soft and hard tissues to a certain extent.

M1-type macrophages, also known as classical activated macrophages (CAMs), stimulate innate immune defense responses and acquired immune responses. They can be activated by microbial stimuli such as lipopolysaccharide (LPS) and interferon-γ (IFN-γ) through classical pathways, as well as by microRNAs (miRNAs), granulocyte macrophage colony stimulating factor (GM-CSF), reactive oxygen species (ROS) and other stimuli. M1 highly expresses CD86 and produce interleukins, such as IL-1β, IL-6, IL-12, and IL-23, as well as other inflammatory cytokines, such as TNF-α, CC chemokine ligand 2 (CCL2), and intercellular adhesion molecule-1 (ICAM-1) (45, 46). They also express proteases, such as inducible nitric oxide synthase (iNOS), MMP-1, MMP-2, cyclooxygenase-2 (COX-2), etc. M1 metabolizes arachidonic acid (AA) to produce PGE2 under the action of COX (**Table 1**). M1 primarily induces a Th1 type immune response. T helper 1 cells (Th1) and resident memory T helper 17 cells (Th17) are closely related to proinflammatory cytokines, such as IL-1, IL-2, TNF-α and IFN-γ, which can increase the effect of the inflammatory response (**Figure 2**). M1 can also phagocytose microorganisms and matrix debris, exhibiting a high antigen presentation capacity during the early healing phase (24).

In periodontal tissue, macrophage polarization to the M1 type is primarily induced by Th1 production of IFN-γ and LPS-dominated microbial-related factors (6). Periodontal pathogenic bacteria can stimulate CD14, Toll-like receptors (TLRs) and NOD-like receptors (NLRs) on the surface of macrophages through LPS, inducing macrophages to differentiate into the M1 type. The secretion of proinflammatory molecules from M1 is regulated by the activation of nuclear factor κ (NF-κB) in activated B cells, activated protein 1 (AP-1) and interferon regulatory factors (IRFs) (45). The contribution of macrophage surface receptors to M1 phenotypic polarization is briefly described below.

TLR2/4 are very important for Porphyromonas gingivalis (P.g.) to promote the polarization of macrophages toward the M1 type (47). In a study by Holden et al. on Porphyromonas gulae (P. gulae) (48), addition of P. gluae promoted the expression of CD86 on the surface of macrophages, and only IFN-γ-activated macrophages (i.e., M1) could secrete nitric oxide (NO) through TLR2 Expression of cytokines was analyzed, and secretion of IL-6 and TNF-α depends on TLR2 and TLR4, respectively. Their findings also suggested that TLR2 has a microbial stimulation effect and that the activation of macrophages plays a nonnegligible role in this process. Recent cytological studies (49, 50) have shown that P.g. is dependent on TLR2 and TLR4 for promoting the secretion of TNF-α in macrophages and is primarily stimulated by glycine lipids, such as serine-glycine dipeptide lipids, of the monomonas factor. Stimulation of TLR2 can weaken the anti-inflammatory activity of M2-like macrophages and produce a chimeric M1/M2 phenotype (51), suggesting that TLR2 is an important receptor for the expression of macrophage proinflammatory functions.

Raudales et al. (52) demonstrated that NLRP3 and ASC gene-deficient macrophages could not be activated by dental calculus, NLRP3-deficient macrophages could not produce IL-1β, and NLR receptors and ASCs were inflammasome-related factors, suggesting that NLRP3 and ASC are involved in the expression of IL-1β in M1. However, Souza et al. found in their study in NOD1-deficient mouse macrophages that after stimulation, the expression of proinflammatory mediators was higher than that of wild type mouse macrophages, accompanied by an increase in the number of osteoclasts, suggesting that NOD1 exerts a bone-protective effect (53), indicating that not all of the receptors in the NLR family are positive inflammation-regulating receptors. The M1 phenotype is also closely related to bone resorption.

The loosening and shedding of teeth is a serious clinical symptom of PD, and the pathological manifestation includes the absorption and destruction of alveolar bone. M1 assists in osteoclast activation by secreting cytokines that promote the Th1 response and stimulate osteoclast precursors. Meanwhile, M1 participates in the expression of cytokines, such as PGE2, IL-1β, TNF-α, IL-6 and IL-12, which are considered to be crucial players in the progression of PD and bone absorption. Among them, PGE2 is the most powerful stimulating factor of periodontal bone resorption, mediating various destruction processes of alveolar bone and cementum, such as reducing the mineralization and survival ability of osteoblasts, mediating the formation of osteoclasts, and stimulating the transformation of cementoblasts into cementum cells (54, 55). LPS is an activator of IL-1β expression in M1, and IL-1β and TNF-α can also activate M1 to produce IL-1β, promoting the activation and differentiation of osteoclasts and ultimately causing bone resorption (56); TNF-α can also induce RANKL production by T cells and B cells (32). IL-1β and IL-6 secreted by M1 induce the expression of MMPs in human gingival fibroblasts (HGFs) and cause the destruction of gingival collagen fibers in inflamed periodontal tissue in high glucose conditions (57). In addition, IL-1β may upregulate the response of HGFs to IL-6, suggesting that IL-1β and IL-6 play a synergistic role in the progression of PD (58). IL-6 can induce osteoclasts to produce MMPs and degrade the extracellular matrix, eventually leading to alveolar bone resorption (59). A study in a mouse model of inflammatory bone destruction revealed that an IL-6 receptor antibody blocked bone resorption and reduced periodontal inflammation (60, 61). MMP-2 can be directly synthesized and secreted by M1, resulting in the destruction and degradation of periodontal connective tissue, which is the primary invader of periodontal tissue destruction (62). Cytokines such as IL-1β, IL-6 and TNF-α produced by activated macrophages and other innate immune cells are known as senescence-associated secretory phenotype (SASP). SASP secretion is increased in senescent cells, while M1 phenotype is also increased in high glucose environment. These associations provide a potential explanation for diabetes and periodontitis, two closely related diseases with age-related morbidity (63).

These studies indicate that M1 plays an important role in inducing bone tissue absorption during the inflammatory response in PD, suggesting that immunotherapy may be able to stop bone tissue absorption in the treatment of PD. However, M1-related cytokine IL-12 Inhibits RANKL and TNF-α induced osteoclasts differentiation; IFN-γ can inhibit RANKL and TNF-α induced osteoclasts differentiation, stimulates osteoclasts apoptosis (64). In a mouse model of periodontitis, Yamaguch et al. (65) also suggested that IFN-γ and IL-12 are responsible for the osteoclastogenesis suppression by M1. Il-27 can induce M1 polarization and inhibit IL-17-mediated Th17 differentiation (66). It should be noted that appropriate functional activity of osteoclasts is also necessary for bone regeneration, and CCL2 and VEGF secreted by M1 also have an obvious recruitment effect on bone marrow mesenchymal stem cells (67). There is no doubt that M1 plays a defensive and destructive role in periodontal hard and soft tissues. Both bacteria and Th1 play an important role in the induction and function of M1. A more specific understanding of the role, timing and primary and secondary differences in the regulation of the various factors is necessary for our in-depth understanding of M1. The following is another important way in which macrophages destroy tissues.

Th17 cells are closely related to M1 and can proliferate and differentiate in chronic periodontal inflammation, representing a key cell type that resists extracellular pathogens and playing a role in promoting the development of inflammation and recruiting neutrophils, characterized by the production of IL-17. In the study of atherosclerosis, Th17 was not mainly induced by T-cell-derived IL-β, but by the proliferation of NETs-mediated macrophage IL-1/IL-17 cascade, which also provides an explanation for the mechanism of neutrophils in maintaining Th17 in chronic aseptic inflammation (68). Current studies have found that Th17 cells in periodontal tissues have different regulation modes in pathological and healthy conditions (8). Aggregation of Th17 cells and their associated neutrophils is a necessary condition for tissue destruction in experimental PD, while the proliferation of Th17 cells requires the stimulation of cytokines such as IL-6, TGF-β, IL-1β, IL-12 and local microorganisms expressed by M1 and maintains the phenotype through IL-23 (69, 70). PD is related to an increase in local IL-17 levels, and the levels of Th17 lymphocytes and IL-17 are positively correlated with the severity of periodontal disease and the clinical parameters of periodontal destruction (71). In addition, the mechanism of IL-17 is relatively complex and plays both an important protective and destructive role in PD. Loss of the IL-17 receptor increases the susceptibility of bacteria to inducing inflammation and periodontal bone loss, so a specific IL-17 receptor signaling pathway is needed to protect bacteria in periodontal tissue. However, excessive IL-17 signaling can lead to inflammation, increasing the production of RANKL in osteoblasts and periodontal ligament fibroblasts, which in turn promotes over-activation of osteoclasts and leads to periodontal bone loss (72, 73); Th17 cell defects are associated with reduced periodontal inflammation and bone loss (74).

The above findings indicate that the activation of M1 participates in defense and inflammatory development, as well as in the regulation of immune cell functions and mechanisms. In recent years, related research has been prolific, and only a small part of this information can be explained here. However, the composition of periodontal tissue is precise, the pathogenicity and interaction of the microbial environment in the periodontal pocket is also very complex, and there are many factors and cells involved in M1. M1 is necessary to build up the network of cells and cytokines associated with periodontal inflammation. The task of identifying a relevant mechanism with clinical application value remains arduous.

### The Functional Role of M2 in Periodontitis

M2-type macrophages, also called alternatively activated macrophages (AAMs), play a relatively weak role in the immune response and primarily function in the process of tissue repair, regeneration and inflammation regression. In addition, M2 is associated with various chronic infections (75). They can be activated by induction of the interleukins IL-4, IL-13, IL-17, etc. (76) and can also be differentiated by stimulation of the immune complex (IC), mesenchymal stem cells (MSCs), glucocorticoids, macrophage-stimulating factor (M-CSF) etc. M2 highly expresses the receptor molecules CD68 and CD206 (namely, the mannose receptor), scavenger receptor, Toll-like receptor, etc., and secrete the anti-inflammatory factors transforming growth factor-β (TGF-β), IL-10, IFN-γ, etc., which play anti-inflammatory and angiogenic roles in promoting tissue repair and wound healing (77); they can also express enzymes, such as argininase (Arg-1). It can also secrete immunomodulatory molecules and chemokines, such as apoptotic protein-1 (ACS-1), CC chemokine ligand 1 (CCL1), and CC chemokine ligand 16 (CCL16) (**Table 1**). M2 is primarily involved in Th2 immunity and regulate the expression of Th2 cells. Th2 cells produce cytokines that promote the humoral immune response, such as IL-4, IL-5, IL-6, IL-10 and IL-13. Simultaneously, TGF-β activates regulatory T cells (Treg cells) to further secrete the anti-inflammatory cytokines TGF-β and IL-10 (**Figure 2**).

In periodontal tissue, M2 plays a role in relieving inflammation and tissue repair, which is characterized by the production of IL-10 and reduced expression of IL-6 (35, 78). Macrophages can differentiate into alternatively activated M2 under stimulation by the Th2-related cytokines IL-4 and IL-13. M2 is considered to exert anti-inflammatory effects due to their ability to inhibit the NF-kB and inflammasome pathways (79, 80). IL-4 can upregulate mannose receptors downregulated by IFN-γ, promoting M2 polarization (81). TGF-β has been considered one of the most important cytokines involved in the maintenance of the M2 phenotype, in part because it inhibits the production of endogenous NO (82); TGF-β is also important for the recruitment of bone MSCs during tissue repair (83). During the development of inflammation, M2 antagonize the response of M1 by producing IL-4, IL-10, IL-13 and TGF-β, regulating anti-inflammatory effects and playing a role in wound healing and tissue repair (84, 85). However, the mechanism by which M2 integrates the molecular signals sensed by IL-4, IL-13 and ACs in apoptotic cells is still unclear. Bosurgi et al. (24) studied a mouse model infected with Neisseria brasiliensis and found that although inductive ACs are essential for anti-inflammatory and tissue repair programs that rely on IL-4 and IL-13 to induce macrophages, they are not necessary for an adaptive type 2 immune response. At the same time, AC signaling and IL-4 can initiate macrophage anti-inflammatory and tissue repair responses in a wide range of environments. In short, IL-4, IL-13 and ACs are the primary driving forces for the polarization of M2 during the process of tissue repair.

IL-10 is an important cytokine involved in the polarization and function of M2 cells. IL-10 transforms immature blood monocytes into M2, directly inhibiting the activity of Th17 cells and in turn enhancing the function of Tregs (86). M2 also expresses high levels of IL-10 (87). In PD, the excessive effects of IL-10 and IL-4 on the healing process seem to be related to the downregulation of proinflammatory cytokines and MMPs and the stimulation of osteoblasts, explaining part of the role of M2 in the formation of new bone after bone resorption (88). On the other hand, IL-10 increases the expression of defense receptor scavenger receptor-A (SR-A) and decreases the expression of CD14, TLR4 and antigen-presenting molecule MHC-II. As the core of LPS-induced cell activation and signal transduction, CD14 and TLR4 play a leading role in the inflammatory response of macrophages to endotoxin. The scavenger receptor (SR) family can be divided into six subfamilies: A, B, C, D, E and F. SR-A has a wide range of ligand-binding activities and interacts with LPS or other bacterial cell wall components to interfere with or weaken CD14-TLR signal transduction. Therefore, it is believed that SR-A not only has a detoxification effect by directly clearing LPS but also has an anti-inflammatory effect (89). In a study of an LSL-K-rasG12D mouse lung cancer model established by Li et al. (90), pretreatment of macrophages with IL-10 effectively inhibited the production of IL-17 by CD4+ T cells, and the M1-Th17 cell axis was the key to tumorigenesis. IL-10 blocks this process primarily through its upstream effect on M1, inducing M1 to M2 transition and inhibiting the activity of Th17 cells.

In addition to IL-10, additional cytokines secreted by M2 also exert anti-inflammatory effects through various mechanisms. Various G protein-coupled receptors (GPCRs) have been shown to activate cAMP production in cells and enhance the expression of M2 markers in different environments, such as the PEG2 receptor, adenosine receptor and atypical chemokine receptor (79, 91). Polumuri et al. (92) reported that increased cAMP enhanced the expression of IL-4-mediated M2 markers in mouse macrophages. In a study of adipose tissue, Luan et al. (91) reported that the paracrine hormone PGE2 enhanced M2-type polarization partly through the activation of cAMP, but the mechanism whereby this occurs remains unclear. M2, memory T cells (8), and NK cells produce IFN-γ, which inhibit the osteoclast activity induced by IL-1 and TNF-α. Meanwhile, M2 produces an interleukin-1 receptor antagonist (IL-1RA) that can block osteoclast activity induced by IL-1 and TNF-α. Additional immunomodulatory molecules secreted by M2, such as CCL1 and CCL16, also regulate the body’s immune response and maintain homeostasis and immune balance (87, 93). M2 accelerates osteogenesis at subsequent stages of implantation by secreting the osteogenesis-related protein BMP-2 (67). IL-34 is able to induce differentiation of circulating monocytes to the M2 phenotype in a manner similar to M-CSF, and GM-CSF and IFN-γ are able to inhibit this effect (94). However, when low-density lipoprotein is elevated, IL-34 increases the expression of SASP in myeloid-derived macrophages and promotes vascular plaque formation (95, 96). We suggest that perhaps further studies of IL-34 may provide ideas for the study of periodontitis in relation to atherosclerosis.

Part of the study above showed that the M2-type polarization can be regulated by M1 and can secrete related factors, and through the adjustment of Th17 and Treg cell function, affecting its anti-inflammatory and repair. The functional recovery of homeostasis after inflammatory injury requires tissue repair and reconstruction of tissue function, whose underlying mechanism is complex and depends on the close interaction between cells to prevent fibrosis or scar formation (97). Previous studies have proved that macrophages play an important coordinating role (98), currently, the role of macrophages in the deinflammatory phase has been precisely identified as the alternating activation phenotype – M2 phenotype (97). M2 also negatively regulates proinflammatory cytokines by secreting repair mediators and anti-inflammatory cytokines, such as IL-4, IL-10, transforming growth factor -β and vascular endothelial growth factor, promoting tissue regeneration and restoration to homeostasis. M2-induced local microenvironment promotes bone integration and angiogenesis, and plays a role in inhibiting bone formation and resorption (99). However, that part of the study was not in the setting of periodontal tissue, and the related mechanism in the other types of tissue inflammation is entirely suitable for PD in the delay process remains to be elucidated.

### Specialized Proresolving Mediator Participates in the Anti-inflammatory Effect of Macrophages

The stage of inflammatory regression, this multistep process includes the cessation of immune cell infiltration and cell engulfment of the PMNs, inhibition of chemokines and cytokines, elimination of apoptotic cells, initiation of tissue repair, and restoration of tissue homeostasis (33, 100). Inflammation resolution is an active receptor-mediated process, specialized pro-resolving mediators (SPMs) are agonists for a number of specific receptors that are naturally involved in inflammation regression through feed-forward mechanisms (101), limit injury, further recruits PMNs, enhance phagocytosis of cellular debris and apoptotic PMN by macrophages, dissolving enzymes that are released to further reduce the PMN, blocking the further development of inflammation (102). In PD, SPMS activates wound healing through tissue regeneration rather than fibrosis or scarring and directly improves bone healing and regeneration (103). SPMs are produced by endogenous polyunsaturated fatty acids (PUFAs) through trans-cellular reactions between human M2 or PMNs and tissues at sites of inflammation (104).

Human macrophages synthesize unique lipid mediator biological signals when stimulated by pathogens. Stimulating M1 can produce arachidonic acid (AA) derivatives, such as leukotriene B4 and PGE2, etc. M2 can be stimulated to produce SPMs, including resolvin D2 (RvD2), RvD5 and maresin-1, and RvD5 can enhance macrophage phagocytosis to a greater extent than leukotriene B4 (105).

The large family of SPMs includes lipoxins (LX) synthesized from AA, E-series resolvins synthesized from eicosapentaenoic acid (EPA), and D-series resolvins, protectins and maresins (MaRs) derived from docosahexaenoic acid (DHA) (106, 107). These lipid mediators can be produced in the human body, LX and MaR are important regulatory factors of macrophage burial (105), and various SPMs perform different functions through specific receptors triggered by G protein-coupled receptors on the surface of macrophages. SPMs negatively regulate proinflammatory mediators, cytokines and prostaglandins (108). MaR was isolated from the exudate during the resolution of mouse peritonitis by Serhan et al. and was demonstrated to be a new pathway for macrophages to synthesize anti-inflammatory mediators from DHA (109). A randomized controlled trial of DHA+ aspirin in patients with moderate to severe periodontitis demonstrated that the combination of DHA+ aspirin can modulated inflammatory response and improve periodontitis (110). MaR has been shown to xert strong activity in promoting the healing of planaria surgical wounds, providing evidence for MaR’s involvement in tissue regeneration and healing (111). Resolution E1 (RvE1) has been demonstrated to promote periodontal regeneration, induce the formation of new alveolar bone and cementum (112, 113), and improve the fibrosis formed in an experimental model of PD (114); Human macrophages exhibit enhanced phagocytosis after contact with RvE1 and appear to promote macrophage growth by inducing phosphorylation of Akt and ribosomal protein S6 (115). Albuquerque-Souza et al. found that both MaR1 and RvE1 restore the regeneration ability of human periodontal ligament stem cells by increasing the activity of human periodontal ligament stem cells, accelerating wound healing/migration, and upregulating periodontal ligament markers and cementoblast differentiation (116). Studying these SPMs is an inspiration for the clinical treatment of PD.

Exposure of M2 to ACs can enhance the expression of anti-inflammatory and tissue repair genes, and its surface liver X receptors (LXRs) play an important role in the clearance of ACs. LXR can coordinate sterol metabolism and immune cell function. Snodgrass et al. (117) found that activation of M2 through stimulation of LXR enhances the ability of cells to synthesize SPM precursors 15-HETE and 17-HDHA, as well as RVD5. When ACS and LXR agonists were exposed together with Th2 cytokines, arachidonate 15-lipoxygenase (ALOX15) and IL-1 receptor antagonist (IL-1RA) selectively enhanced the expression of macrophages. ALOX15 is essential for the synthesis of SPMs (118), and it has been reported that protein levels of ALOX15 directly determine the time of SPM synthesis in human M2 (105) (**Figure 2**).

SPMs promote the recovery of periodontal inflammatory tissue through various mechanisms of action. Clinical studies of SPMs in combination with aspirin did show therapeutic potential, but the intervention sample size was small. SPMs has been proved to have the ability to prevent experimental periodontitis in animal experimental models, such as rats, rabbits, pigs, etc. (119). However, there are few clinical studies at present. Through further research and clinical trials, it may be possible to broaden the treatment options for PD and increase the clinical efficacy.

### Macrophage Polarization and Periodontal Microenvironment

M2 is closely related to periodontal tissue homeostasis and is considered to be an important cell for symbiotic flora to avoid host attack, and has been described as tolerance induction in chronic inflammation stimulated by PAMPs (120, 121). However, the distinction between the concepts of symbiotic and pathogenic bacteria in periodontal tissue has not been fully understood (122). The view of inflammatory diseases such as periodontitis is gradually updated, and it is believed that the microecological imbalance of bacterial community transforms the symbiotic bacterial community into pathogenic bacterial community, and causes periodontal tissue to be in a long-term inflammatory state through continuous immune response (123). Current studies on the prevention and treatment of periodontitis gradually focus on periodontal microbial disorder and tissue homeostasis destruction (124).

Different bacteria can enter the periodontal epithelial barrier in different ways, for example, P.g. can spread through epithelial cells, and A. actinomycetemcomitans (A.a) can move between epithelial cells (125). In the clean-up of P.g., both M1 and M2 showed stronger phagocytosis than macrophages, and M1 infected P.g. After that, a high level of death occurred (126). Other studies have found that P.g. is more likely to combine with M2 than M1 (121, 127). P.g. can also induce M2 polarization, thus avoiding the attack of macrophages (128). Gingipains, an important virality factor produced by P.g, can transform periodontal local complement C5 into C5a, activate C5aR, and weaken the bactericidal function of immune inflammatory cells through contact with TLR2 signal (87), providing opportunities for the invasion of other bacteria (129).When P.g. interferes with the immune response, some of the bacteria that are able to tolerate and exploit the inflammatory environment then behave as pathogenic bacteria (130). Studies on A.a showed that it could enhance the expressions of NLRP3, TLR4, TLR2 and NOD2 in macrophages, but did not enhance the response of human gingival epithelial cells. The mode of immune escape of A.a may be related to the different immune responses of different cells (131). In saliva microbial analysis, it was found that some bacteria in red complex and orange complex were significantly different in proportion between the healthy group and the periodontitis group, suggesting that the type of bacteria is not the most important pathogenic factor, and the change of bacterial abundance may indicate that the characteristics of local immunity in periodontitis have also changed (132). It is very helpful for us to understand the etiology and pathology of periodontitis by studying the local microenvironment of periodontitis and grasping the characteristics of bacteria as a whole to understand the main pathogenic mechanism of bacteria. The study of macrophage polarization may be of positive significance for the reconstruction of tissue homeostasis in periodontal tissues after periodontal treatment, and even the establishment of a healthy ecological flora of individuals.

## Regulatory Mechanisms Based on Polarized Macrophages in Periodontal Tissue

The signaling pathways involved in macrophage polarization in periodontal tissues include MAPK, NF-κB, Akt, Jak/Stat, Notch, Wnt, PPAR-γ, Smad and so on. Various signaling pathways play a variety of roles in the functioning of macrophages in periodontal tissues. Different ligands, receptors, enzymes, transcription factors and other molecules closely cooperate to enable macrophages to exert more precise regulatory ability (133), and different concentrations of the same cytokines can trigger and activate different signaling pathways (134). Key receptors for the polarization of M1-type macrophages include NOD1 and TLR4, which can individually or synergistically activate NF-κB, MAPK and other signaling pathways, triggering the activation and expression of M1 (135). Different SPMs can inhibit M1 polarization or promote M2 polarization to promote the elimination of inflammation through NF-κB, PPAR-γ and other signaling pathways (108, 136). Some of the polarization-related macrophage signaling pathways in periodontal tissue are described below.

### Mitogen-Activated Protein Kinase (MAPK) Signaling Pathway

**Figure 3:**
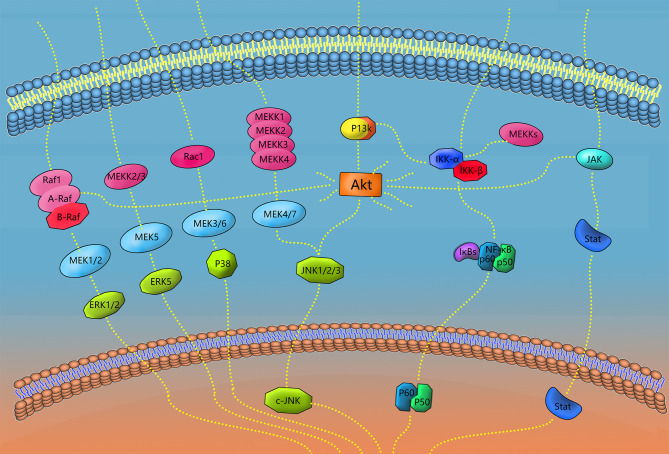
Signaling pathways of polarization and functional expression of periodontal macrophages. In this review, four types of signaling pathways in periodontal macrophages were selected, including MAPK, NF-κB, JAK/STAT, and AKT. When the receptor on the cell membrane is stimulated, the corresponding signaling pathway plays a role, and finally transduces the signal to DNA, transcription and translation of the corresponding effector molecules. A diagram of some links of the signaling pathway mentioned in this paper is shown in the figure.

This pathway signals through the cascade reaction of MAPK series kinases, performs different functions through the four primary branches of JNK, p38/MAPK, ERK and ERK5, and regulates various cellular activities, including proliferation, differentiation and apoptosis (137). Activation of proinflammatory cytokines, such as TNF-α and IL-1β, can induce the JNK and p38/MAPK signaling pathways (90). LPS may increase Ac-LDL uptake and enhance CD204 expression by activating macrophage MAPK/ERK and CD36 expression (138). Xu et al. inhibits RANKL-mediated MAPK pathways (p38, ERK1/2, and JNK) in bone marrow macrophages (also known as osteoclast precursors) in a rat model of PD using chitosan (139). M1-type macrophages can also be polarized by activation of the MAPK signaling pathway alone (140). MAPK pathways (P38, ERK, and JNK) mediate activation of the NLRP3 inflammasome and the secretion of IL-1β and IL-18 (141) (**Figure 3**).

### Janus Kinase (JAK)/Signal Transduction and Transcriptional Activator (STAT) Signaling Pathway

The Janus kinase (JAK)/signal transduction and transcriptional activator (STAT) signaling pathway activates JAK by acting on tyrosine kinase-associated receptors with extracellular signaling molecules as ligands. Activation of DNA has direct effects on signal transduction and transcriptional activation proteins (signal transducer and activator of transcription, STATs) and plays an important role in the pathogenesis of immune-related diseases (142). Among them, IFN-γ is one of the major cytokines involved in M1 polarization, and its receptors recruit JAK1 and JAK2 adapters to activate STAT1, STAT2, and interferon regulatory factor (IRF). IL-12 (IL12A and IL12B) and other pre-M1 genes express IRF through STAT1/STAT2-mediated JAK signaling pathways and actively regulate macrophage activation and polarization (143, 144). When the STAT1 signaling pathway is activated by angiotensin II, macrophage infiltration and proinflammatory cytokine expression are significantly increased in the damaged area of PD, and STAT3 is inhibited (145). Erythropoietin can facilitate M2 polarization through the JAK2/STAT3/STAT6 signaling pathway and has the ability to perform immune regulation in macrophage activation (146). IL-4 and IL-13 activate the JAK3/STAT6 signaling pathway and play an important role in the phenotypic polarization of M2A macrophages. Loss of IL-4 and IL-13 leads to the reduction of M2A phenotypic markers for tissue repair (147). However, IL-4 receptor conjugation of STAT6, which plays an important role in controlling the M2 phenotype, is not sufficient to induce M2 alone during healing, and some studies have reported that M2 is still activated during wound healing in mice with STAT6, IL4-R or IL-10 deletion (11, 148) (**Figure 3**).

### Nuclear Factor-Nuclear Transcription Factor B (NF-κB) Signaling Pathway

This pathway, through TNF and other cytokines, contacts surface receptors, activates IKK kinase, and then dissociates IKB inhibitory protein in NF-κB trimer protein and the dimer formed by NF-κB and p65 or p50, finally exposing the NF-κB dimer to the nuclear localization sequence. It is further transcribed by binding to specific sequences in the nucleus to perform its function. LPS-induced polarization of M1 depends on activation of NF-κBp65, and treatment with IκB kinase-β inhibitors reduces M1-labeled mRNA expression (149). It has been reported that dietary intake of DHA is associated with a reduced prevalence of PD. Choi et al. found that in addition to reducing LPS-induced c-Jun-terminal kinase phosphorylation, DHA also inhibits the transcriptional activity of NF-κB by regulating the nuclear translocation and DNA binding activity of the NF-κBp50 subunit (150). Liu et al. treated LPS-induced experimental PD mice with carbon monoxide releasing molecule-3 (CINM-3) and found that CINM-3 inhibited LPS-induced p-p65, p-p50, and p-IκB expression, inhibited the expression of PD M1-related factors, and increased the expression of M2-related factors (151). Pathak et al. found in ligation-induced PD mice that anti-NF-κB activator 1 (ACT1) mice exhibited higher levels of TNF/NF-κB signal-related p-p65 protein expression, presenting increased PD, alveolar bone loss, macrophage infiltration, inflammation, and M1 polarization (152). NF-κB can be activated by receptor activator of nuclear factor kappa-B ligand (RANKL), which can be effectively upregulated by IL-1β, promoting osteoclast activation and differentiation. The NF signaling pathway can also activate leukocytes to further induce prostaglandin production and cytokine expression (56, 153) (**Figure 3**).

### Phosphatilinositol-3 Kinase (PI3K)/Akt Signaling Pathway

As one of the major signaling pathways regulating macrophages, P13/Akt controls the key switch between immune activation and immunosuppression in the process of inflammation (154), and it participates in the regulation of active and inactive PD. The PI3K/Akt signaling pathway contains a large number of effector molecules upstream and downstream and is characterized by having Akt as the core. Since P13K is the most important upstream molecule of PIP3/PDK/P13K, this signaling pathway is also known as the P13K/Akt signaling pathway. The role of the PI3K/Akt signaling pathway in macrophage polarization has gradually received attention and has been confirmed to mediate the transformation of M2 (155, 156). Guo et al. demonstrated that the absence of GaB1 or GaB2 in Gab family adapters leads to impaired M2 polarization, while IL-4 regulates the polarization of M2 by activating GAB1 to induce the Akt signaling pathway (157). Studying RAW264.7 cells, Wu et al. found that Akt2 overexpression activated the JNK pathway, increasing the release of proinflammatory mediators. In addition, M2 polarization could be promoted by inhibiting Akt2 upstream of JNK. Finally, new Akt2/JNK1/2/c-Jun and Akt2/miR-155–55p/DET1/c-Jun signaling pathways were deduced (as shown in the figure) (158) (**Figure 3**).

The signal pathways related to macrophage polarization are complex and exquisite. Taking expression and activity of serum- and glucocorticoId-inducible kinase 1 (SGK1) as an example, Ren et al. (159) proved that Either IFN-γ/LPS or IL-4/IL-13 phosphorylates SGK1. Activation of SGK1 inhibits the production of M1 inflammatory cytokines, maintains STAT3 levels, and promotes the M2 phenotype; inhibition of SGK1 regulates the increase of the transcription factor Forkhead box-O 1 (FoxO1), and increases the polarization of M1. P13K can activate SGK1 and inhibit FoxO1; FoxO1 is involved in epithelial barrier function, osteoblast apoptosis and dendritic cell presentation ability in other cells (125, 160). Signaling pathways are interconnected and mediate a wide variety of responses between different cell types, which poses challenges to signaling pathway related immunotherapy. Sun et al. (161) used ginsenoside 3 (Rb3) to treat lPS-induced experimental periodontitis rats and found that Rb3 inhibited MAPK/AKT/NF-κB signaling pathway, improved inflammation and reduced alveolar bone resorption in rats. Li et al. (162) found that inhibition of NF-κB and JAK1/STAT1/3 signaling pathway is the main mechanism by which Mangiferin ameliorates experimental periodontitis in mice. Efforts to maintain the balance of the local microenvironment and search for targeted drugs that can regulate the activity of oral pathogens and the function of macrophages are continuing, providing new ideas for the treatment of PD (163).

## Immunoregulatory Treatment of Periodontitis Associated With Macrophage Polarization

The significance of polarized macrophages in the clinical treatment of PD should not be ignored. In addition, specific periodontal tissue complexes contain highly mineralized parenchymal tissue and functional neurovascular interstitial components in parallel, and the study of the control of periodontal inflammation is also of great significance to the field of immune transplantation (164). Inducing the transformation of macrophages to the M2 type in PD to reduce inflammation, promote tissue repair and realize the anti-inflammatory effect is the primary direction of macrophage polarization treatment at present. For instance, Galarraga-Vinueza et al. (165) extracted cranberry concentrate and conducted experiments on LPS-stimulated macrophages (LPS-MAC) and found that M1 polarization was significantly reduced when LPS-MAC were exposed to cranberry concentrate. In contrast, M2 polarization was significantly increased, suggesting that cranberry-derived procyanidins have potential anti-inflammatory effects. It should be noted that antibacterial and alveolar bone protection should be carried out simultaneously. For example, although the absorption of alveolar bone was inhibited in rats with TLR2/TLR4 combined deletion, the systemic transmission of oral bacteria increased (166).

At present, the basic research direction for the treatment of macrophage polarization and PD includes identifying ways to control the polarization of macrophages. Since most treatments for inflammatory diseases, including periodontitis, provide only short-term relief, the new concept of immunomodulatory nanosystems (IMNs) has the potential to change this situation (167). Macrophage polarization is one of the ways in which IMNs play an immunomulatory role. At present, the main IMNs include: 1) engineered nanomaterials like metallic nanoparticles, 2) biologically-derived immuno-potent materials like exosomes, 3) drug delivery platforms, 4) their hybrid combinations. Below, some new technologies and materials that have emerged to regulate the function of macrophages in periodontal tissues are discussed. Due to the limited research on drug delivery platforms in periodontal tissue, we only present 1) and 2) in IMNs.

### Nanomaterials

Inducing macrophage behavior using nanoscale devices to more accurately influence the periodontal microenvironment represents a new direction for periodontal treatment. Antioxidative stress is an important treatment direction of PD (168). Ni et al. (169) induced a change in the macrophage phenotype to the M2 type using 45 nm gold nanoparticles (AuNPs) to regulate the early inflammatory response in periodontal tissue. Thus, a microenvironment was formed that inhibited the levels of inflammatory cytokines and repair cytokines, such as bone morphogenetic protein-2 (BMP-2), promoting periodontal ligament cell differentiation; the secretion of proinflammatory factors in experiment animals was significantly reduced, resulting in the formation of new attachments and cementum. The morphology of the particles can also influence drug utilization by macrophages, Garapaty et al. (170) found that ligands on rods enhanced TNF-α expression compared to spheres. It is important to note that drug release *via* nanocarriers may result in a non-specific immune response due to the location of part of drug release off-target.

In addition to the nanoparticle carrier for treating PD, oral studies that guide macrophage function at the nanoscale have also received much attention. Sun et al. (171) constructed a cerium@Ce6 multifunctional nanocomposite that modulated a reduced M1/M2 ratio to avoid the exacerbation of periodontal inflammation by high levels of reactive oxygen species (ROS) triggered by antimicrobial photodynamic therapy (aPDT), and the regenerative potential of periodontal inflamed tissue was improved in animal models. Wang et al. (172) used the antioxidant drug quercetin onto nano-octahedral ceria to inhibit M1 polarization, promote M2 polarization, and the nanocomposites exhibit scavenging of ROS in an animal model of periodontitis. Wu et al. (173) were able to modify the behavior of macrophages against gingival fibroblasts by modifying the zirconia surface, providing a new method for restoring periodontal soft tissue attachment after repair therapy. In implant-related studies, Yang et al. (174) guided macrophage adhesion by constructing a micro/nanomesh and found that M2 polarization and angiogenesis was promoted.

### Exosomes

Exosomes are small vesicles secreted by various types of cells that are rich in nucleic acids, proteins and lipids. These bioactive substances are internalized and transferred to target cells through fusion and/or endocytosis and play a key role in cell-cell communication in biological processes (175, 176). Exosome is considered to be a key factor in regulating macrophage polarization in various diseases by delivering non-coding RNA, and has gradually shown therapeutic value in studies on myocardial infarction, systemic lupus erythematosus and so on (177, 178); for example, MSC exosomes reduce myocardial ischemia-reperfusion injury in mice by changing macrophage polarization (179). Nakao et al. (180) used TNF-α to enhance exosomes secretion of gingival tissue-derived MSCs in a mouse model of periodontitis, thereby inducing M2 polarization and inhibiting bone loss. Wang et al. (181) examined exosomes secreted using periodical stretch stimulated periodontal ligament cells (PDLs) and their role in macrophage inflammation and found that purified exosomes inhibited IL-1β production in LPS/nigericin-stimulated macrophages. Inhibition of nuclear translocation of NF-κB and the binding activity of NF-κB p65 modulates the homeostasis of the periodontal environment. Dental pulp stem cell-derived exosomes (dPSC-ExOs) modulate macrophage phenotypes, and Shen et al. (182) demonstrated that dPSC-ExO-chitosan hydrogel (dPSC-ExO/CS) accelerated the healing of alveolar bone and periodontal epithelium in mice with PD. Among a variety of molecules that exosomes can contain, miRNA has received more attention. Exosomal miRNA can regulate the phenotype of recipient cells (183), for example, Mir-146a, Mir-125a and Mir-145-5p can regulate the transformation of macrophages from M1 to M2 (184). By cultivating human periodontal ligament tissue, Ning et al. (185) applied microrNA-125A-5P to promote M2 polarization and promote bone healing in orthodontic process. The combination of the ability of miRNA to promote gene expression targeting and the more effective and safe characteristics of exosomes is an important research direction for regulating macrophage polarization and promoting the healing of periodontitis.

### Sustained‐Release Delivery of Drugs

At present, periodontal sustained-release drug delivery systems primarily use films, fibers, gels and nanoparticles as delivery systems (186). The chitosan hydrogel (CS) mentioned above is a reliable controlled drug release material that releases doxycycline, aspirin, antibiotics and other active drugs at the lesion site (187). CS can also be used to load metal particles, cells and exosomes to repair irregularly shaped tissue defects (188). It has also been increasingly applied in periodontiology. Zhuang et al. (189) delivered CCl2 locally with controlled-release microparticles (MPS), increasing the number of M2,and alveolar bone loss was significantly reduced. Local sustained release technology is an important development direction of PD administration. On the one hand, this technology maximizes the pharmaceutical potential of existing PD treatment drugs and increases drug adsorption capacity; on the other hand, it reduces the systemic side effects of drugs and make them safer.

## Conclusions

PD, which occurs due to irreversible alveolar bone resorption and loss of attachment, is a problem that needs to be addressed in various oral treatments. From a cellular point of view, the activation of osteoclasts is the beginning of the progression of gingivitis to PD, and the gradual ascendance of osteoblasts in bone coupling is the beginning of chronic PD. In recent years, the concept of the origin of Macrophages has been gradually updated, while Resident Macrophages and recruited Macrophages have different performances under the effects of aging change and different media stimulation. Further understanding of the origin of macrophages in periodontal tissues will help enlightening the research of periodontitis with the results in other fields. Local periodontal tissue contains multiple mechanisms, coordination and feedback, in which macrophages play an important role as hubs. Macrophage polarization reflects a complex and diverse function and undoubtedly has important implications for the treatment of PD, M1 defense and damage to the immune system, anti-inflammatory repair of the M2 type and interaction using symbiotic bacteria. The mechanisms and signaling pathways are slowly being clarified, and findings indicate the excellent effect of harnessing immune treatment for PD. We believe that the removal of inflammation and tissue protection is one of the keys to the treatment of periodontitis. How to balance or sequential control so that macrophages can remove pathogens, stop inflammation and promote tissue repair will be the focus of drug therapy for periodontitis. Through the in-depth study of the mechanism of action of various cytokines and mediators regulated by macrophage polarization, guiding the proportion of macrophages with different polarization phenotypes to finely control the inflammatory response in periodontal tissue, the therapeutic idea of achieving a good balance between immune defense and tissue homeostasis has been preliminarily demonstrated in some studies.

## Author Contributions

XS made important contributions to the topic selection, content determination and information screening and evaluation of this paper, ensuring the professionalism of this paper and its contribution to periodontal clinical work. JG wrote the article, collected the references and created some of the charts. XM was primarily involved in the revision of articles, structural adjustment and determining logical relationships. XL was involved in the revision of words and sentences, drawing and literature review. LZ screened the reference values of some studies, and RC participated in the reading and collation of the literature. All authors approved the submitted version and agree to be accountable for the content of the work.

## Funding

2020 Disciplinary Construction Project in School of Dentistry(2020kqkyT08); Natural Science Foundation of Anhui Province(KJ2019A0266); Fund Project of Anhui Medical University(2021xkj128); 2021 Disciplinary Construction Project in School of Dentistry, Anhui Medical University (2021kqxkFY15).

## Conflict of Interest

The authors declare that the research was conducted in the absence of any commercial or financial relationships that could be construed as a potential conflict of interest.

## Publisher’s Note

All claims expressed in this article are solely those of the authors and do not necessarily represent those of their affiliated organizations, or those of the publisher, the editors and the reviewers. Any product that may be evaluated in this article, or claim that may be made by its manufacturer, is not guaranteed or endorsed by the publisher.
